# Effects of cognitive rehabilitation training in elderly with mild cognitive impairment a randomized controlled trial

**DOI:** 10.1192/j.eurpsy.2021.1131

**Published:** 2021-08-13

**Authors:** D. Aniwattanapong

**Affiliations:** Psychiatry, Chulalongkorn University, Bangkok, Thailand

**Keywords:** cognitive rehabilitation, CANTAB, mild cognitive impairment, cognitive training

## Abstract

**Introduction:**

Mild cognitive impairment (MCI) becomes increasingly common. It has been demonstrated high risk of progression to dementia. There are no approved medications for treatment of MCI while cognitive intervention might improve cognitive deficits. However, there have been insufficient evidence supporting the effect of the cognitive intervention.

**Objectives:**

To evaluate the effects of a cognitive rehabilitation training in patients with Mild cognitive impairment

**Methods:**

A randomized controlled single-blind trial was conducted. Participants aged ≥ 60 years diagnosed with MCI were recruited and randomly assigned to intervention group (n=32) or waiting list control group (n=32). The intervention was 3-day weekly sessions of multi-component cognitive rehabilitation training for 3 months. Outcomes were assessed by the Cambridge Neuropsychological Test Automated Battery (CANTAB) to measure the effects of intervention at baseline, 3-month and 6-month follow up within the intervention group and compare between intervention group and control group.

**Results:**

The intervention showed significant improvements on the visual episodic memory (p<0.05) and on the executive function (p<0.05) at 3-month follow up. There was a trend towards improvement of cognition between the intervention group and control group, but this effect was not significant. At 6-month follow up, the OTS significantly changed from 3-month follow up, which reflect the maintaining effects of the cognitive training.

**Conclusions:**

The cognitive rehabilitation training has demonstrated improvement of the visual episodic memory and the executive function for the elderly with MCI.

